# Neuropsychiatric Adverse Events with Monoclonal Antibodies Approved for Multiple Myeloma: An Analysis from the FDA Adverse Event Reporting System

**DOI:** 10.3390/ph17101266

**Published:** 2024-09-25

**Authors:** Giuseppe Cicala, Giulia Russo, Vincenza Santoro, Tindara Franchina, Nicola Silvestris, Mariacarmela Santarpia, Edoardo Spina, Maria Antonietta Barbieri

**Affiliations:** 1 Department of Clinical and Experimental Medicine, University of Messina, 98125 Messina, Italy; gcicala@unime.it (G.C.); giuliarusso.ab@gmail.com (G.R.); espina@unime.it (E.S.); 2 Department of Biomedical and Dental Sciences and Morpho-Functional Imaging, University of Messina, 98125 Messina, Italy; vincenza.santoro@unime.it; 3 Department of Human Pathology in Adulthood and Childhood Gaetano Barresi, University of Messina, 98125 Messina, Italy; tfranchina@unime.it (T.F.); nsilvestris@unime.it (N.S.); msantarpia@unime.it (M.S.)

**Keywords:** neuropsychiatric adverse events, multiple myeloma, FAERS, monoclonal antibody, pharmacovigilance

## Abstract

**Background/Objectives**: Monoclonal antibodies (mAbs) have revolutionized multiple myeloma (MM) treatment. However, post-marketing data on their neuropsychiatric safety are limited. This study aimed to evaluate neuropsychiatric adverse events (AEs) related to mAbs used for MM through a retrospective pharmacovigilance analysis using the Food and Drug Administration (FDA) Adverse Events Reporting System (FAERS) database. **Methods**: Individual case safety reports (ICSRs) from 2015 to 2023 with at least one neuropsychiatric AE and one of the MM-approved mAbs as the suspected drug (i.e., daratumumab, elotuzumab, isatuximab, belantamab mafodotin, teclistamab, elranatamab, and talquentamab) were analyzed using descriptive and disproportionality approaches. **Results**: Unknown signals of disproportionate reporting (SDR) included the following: cerebral infarction for daratumumab (*n* = 45; reporting odds ratio (ROR) = 2.39, 95% confidence interval (CI) = 1.79–3.21; information component (IC) = 1.54, IC_025_–IC_075_ = 1.05–1.9), elotuzumab (25; 7.61, 5.13–11.28; 3.03, 2.37–3.51), and isatuximab (10; 2.56, 1.38–4.76; 1.67, 0.59–2.4); mental status changes for daratumumab (40; 2.66, 1.95–3.63; 1.67, 1.14–2.04) and belantamab mafodotin (10; 4.23, 2.28–7.88; 2.3, 1.22–3.03); an altered state of consciousness for daratumumab (32; 1.97, 1.39–2.78; 1.32, 0.73–1.74) and belantamab mafodotin (6; 2.35, 1.05–5.23; 1.6, 0.19–2.52); Guillain-Barre syndrome (GBS) for daratumumab (23; 6.42, 4.26–9.69; 2.81, 2.11–3.3), isatuximab (8; 10.72, 5.35–21.48; 3.57, 2.35–4.37), and elotuzumab (3; 4.74, 1.53–14.7; 2.59, 0.52–3.8); and orthostatic intolerance for daratumumab (10; 12.54, 6.71–23.43; 3.75, 2.67–4.48) and elotuzumab (4; 28.31, 10.58–75.73; 5, 3.24–6.08). **Conclusions**: Our analysis highlighted several previously unacknowledged SDRs for MM-approved mAbs. Given the complex and not entirely understood etiology of some neuropsychiatric AEs, including GBS, further investigations are necessary.

## 1. Introduction

Multiple myeloma (MM) is characterized by the abnormal growth of plasma cells, which produce monoclonal immunoglobulins. This proliferation of cells within the bone marrow frequently leads to bone lesions, kidney damage, anemia, and elevated calcium levels [1]. Monoclonal antibodies (mAbs) have transformed MM treatment, offering significant effectiveness in both newly diagnosed MM (NDMM) and relapsed/refractory MM (RRMM) cases, improving survival rates and treatment compliance while reducing toxicity [2,3]. The five-year overall survival (OS) rates for MM have now surpassed 50% [4]. Daratumumab combined with lenalidomide and dexamethasone extend median OS to 67.6 months compared to 51.8 months with lenalidomide and dexamethasone alone [5]. Elotuzumab improves median progression-free survival (PFS) to 19.4 months [6], while teclistamab shows a median PFS of 11.3 months [7]. By targeting plasma cell antigens, mAbs induce apoptosis through mechanisms such as antibody-dependent cellular cytotoxicity (ADCC), complement-dependent cytotoxicity, inhibition of mitochondrial transfer, and antibody-dependent cellular phagocytosis [8]. MAbs approved by the Food and Drug Administration (FDA) for MM include daratumumab, isatuximab, elotuzumab, belantamab mafodotin (withdrawn from the market), teclistamab, elranatamab, and talquetamab [9,10,11,12,13,14,15].

Although generally well-tolerated, mAbs can cause several adverse events (AEs) [9,10,11,12,13,14,15], including neuropsychiatric ones. While known neuropsychiatric AEs such as neuropathy for daratumumab, elotuzumab, teclistamab, elranatamab, and talquetamab and immune effector cell-associated neurotoxicity syndrome (ICANS) for teclistamab, elranatamab, and talquetamab are documented in the FDA Prescribing Information for these drugs, the literature suggests other undetected potential neuropsychiatric AEs for mAbs. For example, there have been case series reporting leukoencephalopathy and encephalitis with daratumumab [16,17,18] as well as other neurotoxicities, including movement and/or neurocognitive disorders not reported in FDA labels [15,19,20]. However, a comprehensive post-marketing study investigating the neuropsychiatric profile of the new MM therapies is lacking. The present study aims to evaluate and characterize neuropsychiatric AEs related to all mAbs used for MM by analyzing the US FDA Adverse Event Reporting System (FAERS) database to detect new potential neuropsychiatric safety signals.

## 2. Results

### 2.1. Selection Process and Descriptive Analysis

**Figure 1:**
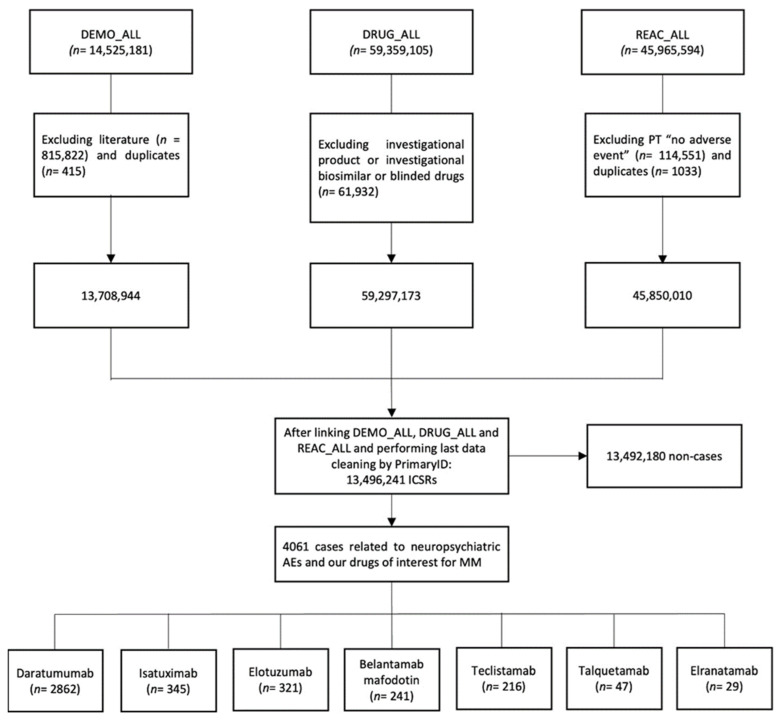
Database cleaning and cases selection flowchart. AE = adverse event; ICSR = individual case safety report; MM = multiple myeloma; and PT = Preferred Term.

After applying the preliminary exclusion criteria and performing the final cleaning of the database, a total of 13,496,241 individual case safety reports (ICSRs) were identified. Among those, 4061 ICSRs met the previously specified inclusion criteria and were classified as cases because they were related to neuropsychiatric AEs and had one of the mAbs approved for MM listed as the suspected drug. Most of these cases (*n* = 2862; 70.5%) were related to daratumumab, followed by isatuximab (*n* = 345; 8.5%) and elotuzumab (*n* = 321; 7.9%) (Figure 1).

Nearly half of the ICSRs were reported for elderly patients (*n* = 1947; 47.9%). This percentage was significantly higher than that observed in the non-cases (*n* = 2,895,017; 21.5%). A higher frequency of male patients was also observed in cases compared to non-cases (*n* = 1849; 45.5% vs. *n* = 4,670,150; 34.6%) (Table 1). A variation in age frequency was noted when stratifying neuropsychiatric ICSRs by each mAb. Specifically, lower frequencies of elderly patients were shown for teclistamab (*n* = 86; 39.8%), belantamab mafodotin (*n* = 69; 28.6%), and talquetamab (*n* = 13; 27.7%) (Table S1). Neuropsychiatric ICSRs were mainly issued by physicians (*n* = 2114; 52.1%), while the main geographical area of reporting was Europe (*n* = 1668; 41.1%). In terms of codified outcomes, neuropsychiatric ICSRs were mainly deemed to be linked to AEs of medical importance (*n* = 1801; 44.4%), followed by AEs leading to or prolonging hospitalization (*n* = 1397; 34.4%). Additionally, 351 ICSRs (8.6%) reported death as an outcome (Table 1). Considering neuropsychiatric AEs by each mAb, belantamab mafodotin and teclistamab-related ICSRs presented higher frequencies of death (*n* = 52; 21.6% and *n* = 42; 19.4%, respectively) (Table S1).

**Figure 2:**
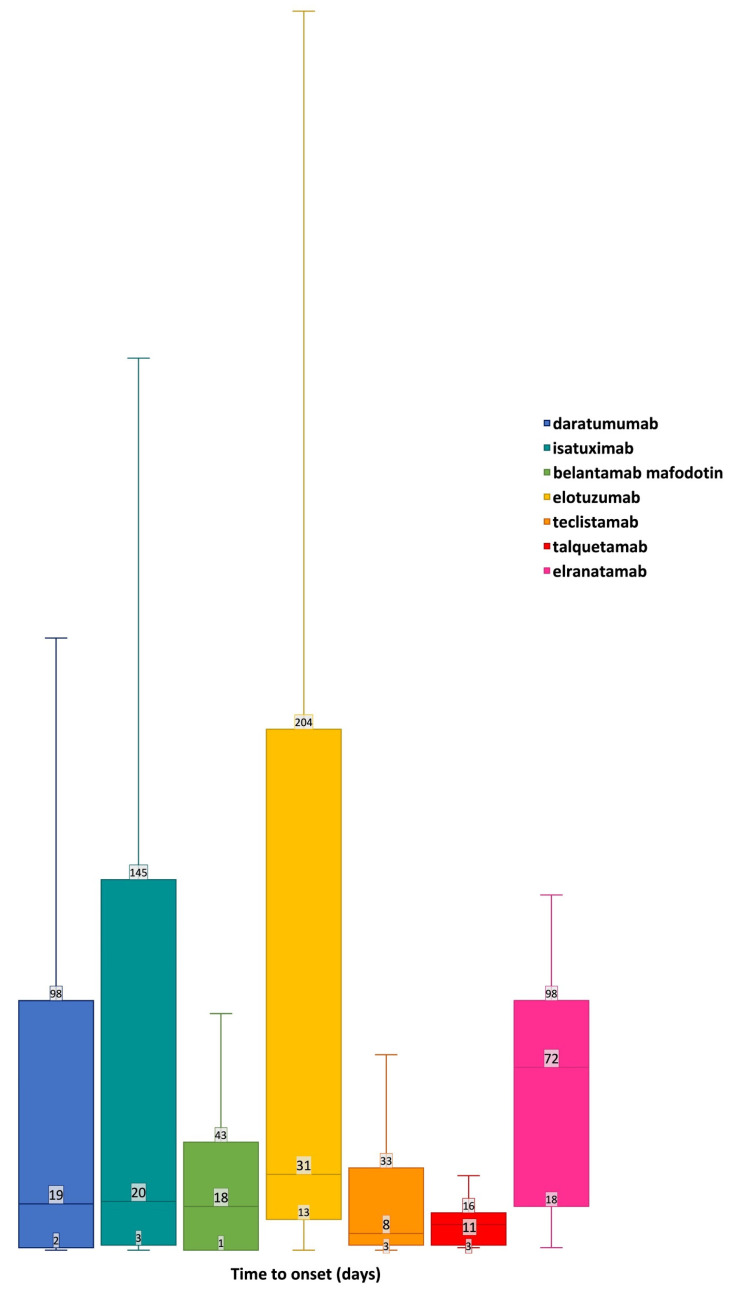
Time to onset of neuropsychiatric AEs. The data are sorted in descending order of frequency and presented as a box plot, with the box extending from the first quartile (Q1) to the third quartile (Q3) and a horizontal line in the middle representing the median time to onset (TTO).

The shortest median (Q1–Q3) time to onset (TTO) for neuropsychiatric AEs was observed with teclistamab at 8 (3–11) days, while the highest median (Q1–Q3) TTO was observed with elranatamab at 72 (18–98) days (Figure 2).

### 2.2. Disproportionality Analysis

New and previously undetected signals of disproportionate reporting (SDRs) using neuropsychiatric AEs were detected by calculating the Reporting Odds Ratios (ROR) and their 95% confidence intervals (CI). The Bayesian information component (IC) was also computed to gauge the association strength between mAbs and AEs. Unexpected AEs were considered as such if not listed in the FDA Prescribing Information. Further details are provided in the Section 4.

Several already acknowledged AEs related to mAbs approved for the treatment of MM emerged as SDRs from our analysis. These included syncope for daratumumab, ICANS for both talquetamab and teclistamab, and peripheral neuropathy for both elotuzumab and elranatamab. The entire disproportionality analysis is available in Table S2. Moreover, some SDRs were linked to other similar known neuropsychiatric AEs. Daratumumab was associated with both polyneuropathies, which could include peripheral sensory neuropathy, and encephalopathies possibly linked to the known posterior reversible encephalopathy syndrome. Elranatamab-related ICSRs reported syncope, with a depressed level of consciousness being a known AE. Furthermore, postherpetic neuralgia, possibly tied to herpes zoster infections, was reported for elotuzumab. However, some unknown AEs also emerged as SDRs (Table 2).

Daratumumab had several SDRs, which included some unknown nervous system-related AEs as follows: cerebral infarction (*n* = 45; ROR = 2.39, 95% CI = 1.79–3.21), a depressed level of consciousness (42; 1.65, 1.22–2.24), ischaemic stroke (33; 2.24, 1.59–3.15; 1.47, 0.89–1.88), an altered state of consciousness (32; 1.97, 1.39–2.78), partial seizures (27; 6.77, 4.63–9.89), spinal cord compression (23; 6.48, 4.29–9.77), Guillain-Barre syndrome (GBS) (23; 6.42, 4.26–9.69), ICANS (18; 5.36, 3.37–8.53), neurotoxicity (17; 1.69, 1.05–2.72), and incoherent (12; 2.61, 1.48–4.61). Considering psychiatric disorders, the AEs not reported in the FDA Prescribing Information for daratumumab were delirium (*n* = 54; ROR = 2.29, 95% CI = 1.75–2.99), mental status changes (40; 2.66, 1.95–3.63), and body dysmorphic disorder (15; 58.08, 34.3–98.33).

Focusing on belantamab mafodotin, unknown SDRs related to nervous system disorders included neuropathy peripheral (*n* = 38; ROR = 2.62, 95% CI = 1.90–3.61), an altered state of consciousness (6; 2.35, 1.05–5.23), muscle tone disorder (4; 59.56, 22.19–159.81), Bell’s palsy (3; 12.77, 4.11–39.68), and neurological decompensation (3; 12.49, 4.02–38.8). Moreover, regarding psychiatric disorders, the only unknown SDR was mental status changes (10; 4.23, 2.28–7.88).

Undocumented nervous system disorders for isatuximab that emerged as SDRs in our analysis included polyneuropathy (*n* = 21; ROR = 9.26, 95% CI = 6.03–14.22), transient ischaemic attack (17; 3.37, 2.09–5.42), ischaemic stroke (14; 4.59, 2.71–7.75), peripheral sensory neuropathy (11; 12.23, 6.76–22.13), cerebral infarction (10; 2.56, 1.38–4.76), cerebral ischaemia (9; 12.64, 6.56–24.34), GBS (8; 10.72, 5.35–21.48), haemorrhage intracranial (7; 2.76, 1.31–5.79), basal ganglia infarction (6; 132.39, 58.54–299.42), peripheral motor neuropathy (6; 29.61, 13.25–66.18), and subarachnoid haemorrhage (5; 2.95, 1.23–7.09). The only unknown SDR for psychiatric disorders was acute psychosis (3; 8.8, 2.83–27.34).

Spinal cord compression was the only unknown neuropsychiatric AE for teclistamab (*n* = 4; ROR = 15.87, 95% CI = 5.94–42.38).

Focusing on elotuzumab, unknown nervous system disorders with SDR included syncope (*n* = 27; ROR = 1.75; 95% CI = 1.2–2.56), cerebral infarction (25; 7.61, 5.13–11.28), cerebral hemorrhage (12; 2.27, 1.29–4; 1.52, 0.54–2.18), cerebrovascular disorder (4; 16.68, 6.24–44.56), orthostatic intolerance (4; 28.31, 10.58–75.73), VI^th^ nerve paralysis (4; 36.99, 13.81–99.05), GBS (3; 4.74, 1.53–14.7), intention tremor (3; 49.87, 15.97–155.8), monoplegia (3; 4.55, 1.46–14.11), and spinal cord compression (3; 4.78, 1.54–14.83). Considering psychiatric disorders, the only unknown SDR was listlessness (3; 5.28, 1.7–16.38). An association between the drug and all unknown AEs was confirmed by the 95% credibility interval limit being greater than 0 for the IC. Further details are available in Table 2.

## 3. Discussion

To the best of our knowledge, this is the first study based on mAb-related neuropsychiatric AEs for the treatment of MM using a large-scale spontaneous reporting system database. Focusing on demographic characteristics, we observed a higher frequency of neuropsychiatric ICSRs involving male patients. The different incidence of MM between male and females might be a key factor in interpreting this result. Male sex is a well-recognized risk factor for the onset of MM. Indeed, a population-based study in the US revealed that, from 2000 to 2019, the age-standardized incidence rates of MM per 100,000 people were 8.49 (95% CI 8.43–8.54) for men and 5.58 (95% CI 5.55–5.62) for women [21]. Literature sources have hypothesized that this increased risk might be related to genetic factors [22]. Additionally, possible lifestyle-dependent risk factors more frequent in male patients (such as smoking or obesity) have also been hypothesized to contribute to the onset of monoclonal gammopathy of undetermined significance, a premalignant precursor to MM [23,24,25]. However, no conclusive evidence exists regarding this in MM at present. Elderly patients were the age category with the highest frequency of neuropsychiatric ICSRs. Over 60% of MM diagnoses in the US are made in patients aged 65 years and older [26]. This might be due to early nonspecific symptoms of MM, such as back pain, fatigue, and anemia, which can often be mistaken for age-related issues, leading to delays in MM diagnosis and treatment [27]. Furthermore, elderly patients are known to be more susceptible to the onset of AEs in general [28,29], and age is also considered a risk factor for the development of neuropsychiatric AEs, such as peripheral neuropathy and polyneuropathy, in MM patients [30].

Serious outcomes, including hospitalization and important medical events, were mainly observed in neuropsychiatric ICSRs compared to the non-case group. The line therapy of mAbs in MM treatment should be considered in this context. Indeed, daratumumab is the only mAb currently approved for NDMM. Thus, a relevant portion of the ICSRs could pertain to patients with RRMM. These patients are typically older, have undergone several lines of previous therapies, and may have disease-related comorbidities [31].

Considering the TTO, elranatamab-related ICSRs exhibited the highest median TTO among all mAbs for neuropsychiatric AEs. Elranatamab-related neuropsychiatric AEs with a longer TTO were mainly associated with alterations in consciousness, such as syncope, a depressed level of consciousness, and an altered state of consciousness. These manifestations have previously been observed as part of cytokine release syndromes [32]. However, these AEs are mostly reported during the step-up phases of treatment, with randomized controlled trial data highlighting a median (Q1–Q3) TTO of 2 (1–9) days [33]. Thus, the observed prolonged TTO might be due to other factors, such as dose delays or interruptions, which could be implemented as mitigation strategies following the onset of previous AEs such as infections or hematologic AEs [33].

The disproportionality analysis highlighted SDRs in vascular disorders involving the central nervous system (CNS). Both cerebral infarction and ischaemic stroke were previously unknown for daratumumab, isatuximab, and elotuzumab. Literature data regarding specific CNS vascular complications in MM patients treated with mAbs are currently lacking. However, pre-marketing safety data for both daratumumab and isatuximab highlighted non-relevant effects on the frequency of vascular thromboembolic events (VTE) in general [34,35]. Other factors might play a key role in the onset of these AEs. Indeed, MM patients are frequently characterized by hypercoagulability states, which could facilitate the onset of VTEs [36,37]. Furthermore, the co-administration of mAbs with immunomodulatory drugs, such as lenalidomide and pomalidomide, represents a well-recognized risk factor for VTEs [38]. Additionally, several disproportional haemorrhage-related AEs were observed, such as cerebellar haemorrhage for daratumumab and intracranial haemorrhage for isatuximab. In these cases, disease progression in MM might play a key role in the onset of these AEs. Indeed, MM patients exhibit the highest incidence of thrombocytopenia among those with haematological cancers, which is a significant risk factor for bleeding [39]. Moreover, dysfibrinogenemia, often observed in MM patients due to interactions between MM paraproteins and coagulation proteins, can also lead to bleeding complications [40,41,42].

AEs associated with alterations in state of consciousness were also identified as unknown SDRs. Specifically, a depressed or altered level of consciousness, an incoherent state, stupor, and a worsening of senile dementia had higher RORs for daratumumab. Alterations in consciousness and mental status were also SDRs for belantamab mafodotin, along with neurological decompensation. Delirium was identified as an SDR for elotuzumab, together with listless. Finally, acute psychotic episodes were unknown SDRs for isatuximab. Altered mental status (AMS) in MM patients is often due to metabolic disturbances such as uremia, hypercalcemia, and hyperviscosity. Elevated levels of serum ammonia have also been reported as a rare but clinically impactful cause of AMS in these patients [43]. Furthermore, a population-based study showed a strong correlation between peripheral neuropathies (PNs) and the degradation of cognitive performance, which could lead to AMS in elderly patients [44]. AMS conditions might also result from the co-administration of immunomodulators, which could themselves be related to neurotoxicity [45]. Moreover, AMS might be a part of more complex clinical issues, such as encephalopathies [46], which were disproportionally reported for daratumumab and elotuzumab. The posterior reversible encephalopathy syndrome is an already-documented AE for daratumumab. This condition is characterized by reversible vasogenic cerebral edema that manifests acutely with neurological symptoms such as seizures, headaches, and visual disturbances, in addition to AMS [47].

Neuropathies were also identified as SDRs in several mAbs. The onset of PNs was not mentioned in the FDA Prescribing Information for isatuximab and belantamab mafodotin. The neuronal damage that could lead to PNs might theoretically be caused by isatuximab and belantamab mafodotin through mechanisms such as ADCC [48,49] and CDC [50]. However, PNs can also emerge as consequences of worsening MM [30,51] due to deposits of the M-protein produced by myeloma cells on neurons [52]. Furthermore, isatuximab is currently approved only as a third-line treatment, while belantamab mafodotin was approved as a fifth-line therapy before it was withdrawn. Thus, compromised patient conditions should be considered as a possible influencing factor [53,54]. Moreover, the concomitant use of pomalidomide and carfilzomib with daratumumab or isatuximab could also be associated with the onset of PNs in MM patients [55,56]. Neuropathies can be associated with both sensory symptoms (e.g., numbness, tingling, pain) and motor symptoms (e.g., muscle weakness). In some cases, PNs can also be associated with paralysis [30]. Our data were in line with this, as unknown VI^th^ nerve paralyses were SDRs for daratumumab, belantamab mafodotin, and elotuzumab. Moreover, a rare severe form of PN, characterized by a rapidly-advancing, symmetrical limb weakness [51,52] and known as GBS, was also disproportionally reported for daratumumab, isatuximab, and elotuzumab [49,50]. The mechanisms underlying the onset of GBS remain unclear; however, the presence of a previous infection is considered an important factor [57]. Although immunodeficiency is a common feature of MM [58,59], both daratumumab and isatuximab-based therapies have been linked to an increased risk of infections [60]. Indeed, the results of a recent meta-analysis showed that among anti-CD38-treated patients, the relative risk for any grade infections compared with the control group was 1.27 (95% CI, 1.17–1.37) [61]. This increased susceptibility to infections could potentially trigger the onset of GBS in predisposed individuals.

### Strengths and Limitations

Spontaneous reporting system database-based analyses are among the most widely used methodologies for generating hypotheses about drug safety in pharmacovigilance [62,63]. The large-scale nature of the FAERS database enables the detection of AEs not previously identified in controlled environment studies [64]. However, some limitations inherent to the chosen methodology are present. The absence of a proper denominator prevents us from determining the incidence of the observed AEs [65]. Additionally, pharmacovigilance databases are mainly based on spontaneous reporting, which can lead to underreporting or overreporting of events due to various external factors [66,67]. Another limitation is the potential presence of duplicate ICSRs. To mitigate this issue, we implemented a multi-step control process based on key information fields, as detailed in the Section 4. Several additional measures were also implemented to improve data quality, such as eliminating undescriptive AEs and using validated data extraction and processing tools as well as a standardized drug naming dictionary [68]. Most of the mAbs considered are prescribed as a second or subsequent line of treatment for patients with RRMM. Therefore, the influence of disease progression on the reporting of neuropsychiatric AEs cannot be excluded. The observed disproportionalities may also have been influenced by the presence of other co-administered drugs, which complicates establishing a causal relationship between the observed AEs and mAbs. Furthermore, the lack of complete patient clinical histories, which are not available in the open FAERS data, limits our ability to conduct a more comprehensive evaluation. Despite these limitations, we believe our study provides valuable insights for oncologists, aiding in the understanding of the neuropsychiatric safety profile of mAbs and assisting in the management of MM patients.

## 4. Materials and Methods

### 4.1. Study Design

A retrospective pharmacovigilance study was conducted to identify neuropsychiatric AEs associated with mAbs approved for MM using the FAERS database. The FAERS database, a widely utilized public resource, has consistently demonstrated its reliability as a platform for drug safety evaluation studies [69,70,71,72]. This database aggregates over 20 million ICSRs from patients, healthcare providers, and pharmaceutical companies across the US, Europe, and Asia. Each ICSR includes a primary ID, data related to the individual (e.g., gender, age, and weight), reporting details such as the reporting country and the qualification of primary sources, information on suspected and concomitant drugs—including their indications and administration dates—and suspected AEs classified by the Medical Dictionary for Regulatory Activities (MedDRA^®^) Preferred Term (PT) [73], along with details on the date of onset and the outcome.

### 4.2. Selection of Cases

Using the zipped ASCII FAERS quarterly data extract files accessible at https://fis.fda.gov/extensions/FPD-QDE-FAERS/FPD-QDE-FAERS.html (accessed on 29 January 2024), we downloaded data from the first quarter (Q1) of 2015 to the fourth quarter (Q4) of 2023, covering the period since the approval of the first mAbs for MM.

In detail, we retrieved data from each DEMO, DRUG, INDI, OUTC, REAC, and THER file. These files were merged based on the primary ID and the case ID. Information from INDI and THER files was combined with DRUG data to create a comprehensive file named DRUG_ALL. Similarly, OUTC data were merged with DEMO data to generate a file renamed DEMO_ALL. Additionally, the REAC_ALL file contained data exclusively from the REAC file.

Each of these three files—DRUG_ALL, DEMO_ALL, and REAC_ALL—was cleaned by removing all duplicated ICSRs based on primary ID and case ID as well as key fields including type of AEs, date of onset, gender, age, reporting country, and suspected drug. This process followed FDA recommendations, wherein in cases with multiple ICSRs sharing the same primary ID, only the most recent case ID version are retained [74].

From the DEMO_ALL file, premarketing ICSRs with supporting literature were excluded. Additionally, for the DRUG_ALL file, we utilized the DiAna dictionary—a dynamic, open-source tool known for its dynamic nature, transparency, and adaptability. This dictionary was used to map all drug names in active substances within each ICSR according to the Anatomical Therapeutic Chemical (ATC) classifications [68]. We also excluded ICSRs that contained at least one investigational product, investigational biosimilar product, or blinded product. Similarly, from the REAC_ALL file, all cases with the PT “no adverse event” were excluded.

For defining our cases, we selected all ICSRs for which one of the following drugs was listed as the primary or secondary suspect: daratumumab, elotuzumab, isatuximab, belantamab mafodotin, teclistamab, elranatamab, and talquentamab. To avoid therapeutic biases, ICSRs with indications other than MM were excluded. Moreover, to analyze neuropsychiatric AEs, we considered all ICSRs containing at least one AE classified under the SOC “nervous system disorders” or “psychiatric disorders”.

### 4.3. Data Analyses

The demographic and clinical characteristics of FAERS ICSRs were analyzed using a descriptive statistical approach with a case–non-case comparison. Continuous variables are presented as medians with quartiles (Q1–Q3), while categorical variables are shown as absolute values with corresponding percentages. Key variables analyzed include gender, age, the primary source of information, year of reporting, reporting country, and detailed descriptions of AEs, including their outcome and the TTO. The TTO was calculated as the interval between the drug administration (start date) and the AE manifestation (event date) and was presented as a median (Q1–Q3) for clarity.

A disproportionality analysis was conducted to detect new and previously undetected SDRs for neuropsychiatric PTs by calculating the ROR and its 95% CI. Statistical significance was determined if the lower limit of the 95% CI for the ROR was greater than one, with a minimum of three ICSRs for each drug-event combination [70].

To reduce the risk of identifying spurious associations and to assess the strength of the association between mAbs and AEs, the Bayesian IC was computed. An association between the drug and the AE was indicated by a 95% credibility interval limit greater than 0 (IC_025_ > 0). AEs not listed in the FDA Full Prescribing Information for each mAb at the time of this study were considered unexpected [9,10,11,12,13,14,15].

The significance level for statistical analyses was set at a *p* value < 0.05. All data processing and statistical analyses were conducted using R (version 4.3.1) with the RStudio (version 2024.04.2+764) [75,76].

## 5. Conclusions

This study underscores the crucial role of large-scale spontaneous reporting system databases in evaluating AEs. Our findings are consistent with the limited existing literature on neuropsychiatric AEs associated with mAbs used in the treatment of MM. We identified several previously unrecognized neuropsychiatric AEs related to mAbs, including VTE, AMS, and GBS. Further research is needed to better understand and contextualize these tolerability issues. Additionally, our study highlights the importance of the ongoing monitoring of MM patients for neuropsychiatric AEs. Timely management of these AEs can enhance patient quality of life and, in some cases, such as alterations in consciousness, may help reduce the impact of associated complications.

## Figures and Tables

**Table 1:** Characteristics of cases related to neuropsychiatric adverse events of monoclonal antibodies approved for multiple myeloma compared to non-cases.

Characteristic	Neuropsychiatric Cases (*n* = 4061)	Non-Cases (*n* = 13,492,180)	Total (*n* = 13,496,241)
Age group, *n* (%)			
Neonate	3 (<0.1%)	39,379 (0.3%)	39,382 (0.3%)
Infants		15,400 (0.1%)	15,400 (0.1%)
Child	7 (0.2%)	150,436 (1.1%)	150,443 (1.1%)
Adolescent	10 (0.3%)	196,410 (1.5%)	196,420 (1.5%)
Adult	1006 (24.8%)	4,157,969 (30.8%)	4,158,975 (30.8%)
Elderly	1947 (47.9%)	2,895,017 (21.5%)	2,896,964 (21.5%)
Not available	1088 (26.8%)	6,037,569 (44.8%)	6,038,657 (44.7%)
Sex, *n* (%)			
Female	1588 (39.1%)	7,145,404 (53.0%)	7,146,992 (53.0%)
Male	1849 (45.5%)	4,670,150 (34.6%)	4,671,999 (34.6%)
Not available	624 (15.4%)	1,676,626 (12.4%)	1,677,250 (12.4%)
Primary source qualification, *n* (%)			
Consumers	580 (14.3%)	6,659,308 (49.4%)	6,659,888 (49.4%)
Health professional	685 (16.9%)	1,185,741 (8.8%)	1,186,426 (8.8%)
Physician	2114 (52.1%)	2,801,856 (20.8%)	2,803,970 (20.8%)
Other health-professional	352 (8.7%)	1,140,098 (8.5%)	1,140,450 (8.5%)
Pharmacist	312 (7.7%)	884,427 (6.6%)	884,739 (6.6%)
Lawyer		502,463 (3.7%)	502,463 (3.7%)
Not available	18 (0.4%)	318,287 (2.4%)	318,305 (2.4%)
Outcome codification, *n* (%)			
Death	351 (8.6%)	780,158 (5.8%)	780,509 (5.8%)
Disability	69 (1.7%)	147,322 (1.1%)	147,391 (1.1%)
Hospitalization—Initial or prolonged	1397 (34.4%)	2,090,657 (15.5%)	2,092,054 (15.5%)
Life-threatening	112 (2.8%)	143,135 (1.1%)	143,247 (1.1%)
Other serious (Important Medical Event)	1801 (44.4%)	4,233,022 (31.4%)	4,234,823 (31.4%)
Required intervention to prevent permanent impairment/damage	5 (0.1%)	12,674 (0.1%)	12,679 (0.1%)
Congenital anomaly		21,535 (0.2%)	21,535 (0.2%)
Not available	326 (8.0%)	6,063,677 (44.9%)	6,064,003 (44.9%)
Reporter Country, *n* (%)			
Africa	19 (0.5%)	37,622 (0.3%)	37,641 (0.3%)
Asia	635 (15.6%)	667,858 (5.0%)	668,493 (5.0%)
Central America	18 (0.4%)	28,633 (0.2%)	28,651 (0.2%)
Europe	1668 (41.1%)	1,749,620 (13.0%)	1,751,288 (13.0%)
North America	1414 (34.8%)	10,100,868 (74.9%)	10,102,282 (74.9%)
Oceania	77 (1.9%)	95,927 (0.7%)	96,004 (0.7%)
South America	172 (4.2%)	227,114 (1.7%)	227,286 (1.7%)
Not available	58 (1.4%)	584,538 (4.3%)	584,596 (4.3%)
Year of reporting, *n* (%)			
2015	17 (0.4%)	1,239,483 (9.2%)	1,239,500 (9.2%)
2016	251 (6.2%)	1,300,142 (9.6%)	1,300,393 (9.6%)
2017	250 (6.2%)	1,356,259 (10.1%)	1,356,509 (10.1%)
2018	414 (10.2%)	1,616,069 (12.0%)	1,616,483 (12.0%)
2019	471 (11.6%)	1,628,852 (12.1%)	1,629,323 (12.1%)
2020	469 (11.6%)	1,681,724 (12.5%)	1,682,193 (12.5%)
2021	535 (13.2%)	1,706,194 (12.7%)	1,706,729 (12.7%)
2022	748 (18.4%)	1,628,953 (12.1%)	1,629,701 (12.1%)
2023	906 (22.3%)	1,334,504 (9.9%)	1,335,410 (9.9%)
Median age (Q1–Q3), years	69 (61–75)	60 (44–71)	60 (44–71)
Median weights (Q1–Q3), Kgs	70 (60–85)	73 (60–88)	73 (60–88)

**Table 2:** Disproportionality analyses and notoriety evaluations based on the Food and Drug Administration Prescribing Information for neuropsychiatric adverse events related to monoclonal antibodies approved for multiple myeloma.

**Daratumumab**					
**SOC**	**PT**	**N**	**ROR (95% CI)**	**IC (IC_025_–IC_075_)**	**Expected in FDA Prescribing Information**
Nervous system disorders	Neuropathy peripheral	533	5.89 (5.4–6.42)	2.64 (2.49–2.74)	Uk (peripheral sensory neuropathy)
	Polyneuropathy	189	17.74 (15.34–20.5)	4.15 (3.91–4.32)	Uk (peripheral sensory neuropathy)
	Syncope	145	1.66 (1.41–1.95)	1.11 (0.83–1.31)	Yes
	Encephalopathy	71	5.07 (4.01–6.41)	2.48 (2.08–2.76)	Uk (posterior reversible encephalopathy syndrome)
	Peripheral sensory neuropathy	45	10.49 (7.81–14.08)	3.45 (2.96–3.81)	Yes
	Cerebral infarction	45	2.39 (1.79–3.21)	1.54 (1.05–1.9)	No
	Depressed level of consciousness	42	1.65 (1.22–2.24)	1.12 (0.6–1.48)	No
	Ischaemic stroke	33	2.24 (1.59–3.15)	1.47 (0.89–1.88)	No
	Altered state of consciousness	32	1.97 (1.39–2.78)	1.32 (0.73–1.74)	No
	Presyncope	32	1.42 (1–2.01)	0.96 (0.37–1.37)	Yes
	Posterior reversible encephalopathy syndrome	29	6.13 (4.25–8.84)	2.74 (2.12–3.18)	Yes
	Nervous system disorder	28	1.69 (1.16–2.45)	1.15 (0.52–1.59)	Yes
	Partial seizures	27	6.77 (4.63–9.89)	2.87 (2.23–3.33)	No
	Leukoencephalopathy	26	14.8 (10.04–21.84)	3.93 (3.28–4.4)	Uk (posterior reversible encephalopathy syndrome)
	Spinal cord compression	23	6.48 (4.29–9.77)	2.82 (2.12–3.31)	No
	Guillain-Barre syndrome	23	6.42 (4.26–9.69)	2.81 (2.11–3.3)	No
	Brain oedema	20	2.51 (1.62–3.9)	1.62 (0.87–2.14)	Uk (peripheral oedema)
	Facial paralysis	19	1.6 (1.02–2.52)	1.1 (0.33–1.64)	Uk (peripheral sensory neuropathy)
	Peripheral sensorimotor neuropathy	18	22.42 (14.02–35.85)	4.51 (3.72–5.07)	Uk (peripheral sensory neuropathy)
	ICANS	18	5.36 (3.37–8.53)	2.58 (1.79–3.13)	No
	Neurotoxicity	17	1.69 (1.05–2.72)	1.16 (0.35–1.73)	No
	Peripheral motor neuropathy	14	14.48 (8.53–24.58)	3.93 (3.02–4.55)	Uk (peripheral sensory neuropathy)
	Incoherent	12	2.61 (1.48–4.61)	1.68 (0.71–2.35)	No
	Orthostatic intolerance	10	12.54 (6.71–23.43)	3.75 (2.67–4.48)	No
	Stupor	8	4.48 (2.23–8.98)	2.39 (1.18–3.19)	No
	Senile dementia	6	10.24 (4.57–22.93)	3.52 (2.1–4.43)	No
	Intracranial mass	6	4.61 (2.07–10.3)	2.45 (1.04–3.36)	No
	Cytotoxic oedema	5	41.17 (16.71–101.45)	5.44 (3.88–6.42)	Uk (peripheral oedema)
	Allodynia	5	8.64 (3.57–20.86)	3.31 (1.74–4.29)	Uk (nerve damage causing tingling, numbness or pain)
	Hyperammonaemic encephalopathy	5	7.57 (3.13–18.26)	3.13 (1.57–4.11)	Uk (posterior reversible encephalopathy syndrome)
	Paraparesis	5	3.69 (1.53–8.89)	2.18 (0.62–3.17)	Uk (peripheral sensory neuropathy)
	Pleocytosis	4	11.42 (4.25–30.68)	3.72 (1.95–4.8)	No
	VI^th^ nerve paralysis	4	6.48 (2.42–17.35)	2.95 (1.19–4.03)	Uk (nerve damage causing tingling, numbness or pain)
	Cerebellar haemorrhage	4	3.04 (1.14–8.11)	1.97 (0.2–3.04)	No
	Loss of proprioception	3	12.5 (3.99–39.14)	3.89 (1.82–5.1)	No
	Cerebellar haematoma	3	11.42 (3.65–35.74)	3.77 (1.7–4.98)	No
	Toxic neuropathy	3	10.67 (3.41–33.38)	3.68 (1.61–4.88)	Uk (peripheral sensory neuropathy)
	Autonomic neuropathy	3	3.93 (1.26–12.21)	2.34 (0.27–3.55)	Uk (peripheral sensory neuropathy)
Psychiatric disorders	Delirium	54	2.29 (1.75–2.99)	1.49 (1.04–1.81)	No
	Mental status changes	40	2.66 (1.95–3.63)	1.67 (1.14–2.04)	No
	Body dysmorphic disorder	15	58.08 (34.3–98.33)	5.81 (4.94–6.41)	No
	Anxiety disorder	12	3.6 (2.04–6.35)	2.08 (1.1–2.75)	Yes
**Belantamab Mafodotin**					
**SOC**	**PT**	**N**	**ROR (95% CI)**	**IC (IC_025_–IC_075_)**	**Expected in FDA Prescribing Information**
Nervous system disorders	Neuropathy peripheral	38	2.62 (1.9–3.61)	1.65 (1.11–2.03)	No
	Altered state of consciousness	6	2.35 (1.05–5.23)	1.6 (0.19–2.52)	No
	Muscle tone disorder	4	59.56 (22.19–159.81)	6.06 (4.29–7.14)	No
	Bell’s palsy	3	12.77 (4.11–39.68)	3.94 (1.87–5.15)	No
	Neurological decompensation	3	12.49 (4.02–38.8)	3.91 (1.84–5.12)	No
Psychiatric disorders	Mental status changes	10	4.23 (2.28–7.88)	2.3 (1.22–3.03)	No
**Elranatamab**					
**SOC**	**PT**	**N**	**ROR (95% CI)**	**IC (IC_025_–IC_075_)**	**Expected in FDA Prescribing Information**
Nervous system disorders	Altered state of consciousness	3	20.7 (6.61–64.83)	4.6 (2.53–5.81)	Yes
	Syncope	3	3.82 (1.22–11.98)	2.29 (0.22–3.5)	Uk (depressed level of consciousness)
	Neuropathy peripheral	3	3.6 (1.15–11.28)	2.22 (0.15–3.42)	Yes
**Isatuximab**					
**SOC**	**PT**	**N**	**ROR (95% CI)**	**IC (IC_025_–IC_075_)**	**Expected in FDA Prescribing Information**
Nervous system disorders	Polyneuropathy	21	9.26 (6.03–14.22)	3.31 (2.58–3.82)	No
	Transient ischaemic attack	17	3.37 (2.09–5.42)	1.98 (1.17–2.55)	No
	Ischaemic stroke	14	4.59 (2.71–7.75)	2.39 (1.48–3.01)	No
	Peripheral sensory neuropathy	11	12.23 (6.76–22.13)	3.72 (2.7–4.42)	No
	Cerebral infarction	10	2.56 (1.38–4.76)	1.67 (0.59–2.4)	No
	Cerebral ischaemia	9	12.64 (6.56–24.34)	3.78 (2.64–4.54)	No
	Guillain-Barre syndrome	8	10.72 (5.35–21.48)	3.57 (2.35–4.37)	No
	Haemorrhage intracranial	7	2.76 (1.31–5.79)	1.79 (0.48–2.64)	No
	Basal ganglia infarction	6	132.39 (58.54–299.42)	7.11 (5.7–8.02)	No
	Peripheral motor neuropathy	6	29.61 (13.25–66.18)	5.01 (3.6–5.92)	No
	Subarachnoid haemorrhage	5	2.95 (1.23–7.09)	1.9 (0.34–2.89)	No
	Acute motor-sensory axonal neuropathy	4	93.02 (34.43–251.26)	6.68 (4.91–7.76)	No
	Meningoradiculitis	3	179.87 (56.31–574.58)	7.64 (5.57–8.85)	No
	Chronic inflammatory demyelinating polyradiculoneuropathy	3	11.95 (3.84–37.14)	3.85 (1.78–5.05)	No
Psychiatric disorders	Acute psychosis	3	8.8 (2.83–27.34)	3.42 (1.36–4.63)	No
**Talquetamab**					
**SOC**	**PT**	**N**	**ROR (95% CI)**	**IC (IC_025_–IC_075_)**	**Expected in FDA Prescribing Information**
Nervous system disorders	Dysgeusia	13	17.71 (10.11–31.02)	4.16 (3.22–4.8)	Yes
	ICANS	7	185.55 (87.3–394.35)	7.59 (6.29–8.44)	Yes
	Taste disorder	7	26.81 (12.63–56.93)	4.82 (3.52–5.68)	Yes
	Ageusia	5	18.81 (7.75–45.66)	4.37 (2.81–5.36)	Yes
	Neurotoxicity	3	26.11 (8.35–81.62)	4.93 (2.86–6.14)	Yes
**Teclistamab**					
**SOC**	**PT**	**N**	**ROR (95% CI)**	**IC (IC_025_–IC_075_)**	**Expected in FDA Prescribing Information**
Nervous system disorders	ICANS	96	450.7 (364.76–556.89)	8.66 (8.32–8.9)	Yes
	Neurotoxicity	22	31.49 (20.65–48.02)	5 (4.29–5.51)	Yes
	Polyneuropathy	5	6.45 (2.68–15.53)	2.92 (1.36–3.91)	Yes
	Nervous system disorder	5	4.27 (1.78–10.28)	2.37 (0.81–3.36)	Yes
	Depressed level of consciousness	5	2.79 (1.16–6.71)	1.83 (0.27–2.82)	Yes
	Spinal cord compression	4	15.87 (5.94–42.38)	4.19 (2.43–5.27)	No
	Encephalopathy	4	4.02 (1.51–10.72)	2.32 (0.56–3.4)	Yes
	Unresponsive to stimuli	4	3.44 (1.29–9.19)	2.13 (0.36–3.21)	Uk (depressed level of consciousness)
Psychiatric disorders	Mental status changes	5	4.7 (1.95–11.31)	2.5 (0.94–3.48)	Yes
**Elotuzumab**					
**SOC**	**PT**	**N**	**ROR (95% CI)**	**IC (IC_025_–IC_075_)**	**Expected in FDA Prescribing Information**
Nervous system disorders	Neuropathy peripheral	41	2.52 (1.85–3.43)	1.6 (1.08–1.97)	Yes
	Syncope	27	1.75 (1.2–2.56)	1.19 (0.55–1.64)	No
	Cerebral infarction	25	7.61 (5.13–11.28)	3.03 (2.37–3.51)	No
	Cerebral haemorrhage	12	2.27 (1.29–4)	1.52 (0.54–2.18)	No
	Cerebrovascular disorder	4	16.68 (6.24–44.56)	4.26 (2.5–5.34)	No
	Clumsiness	4	7.82 (2.93–20.86)	3.21 (1.45–4.29)	Uk (peripheral motor neuropathy)
	Orthostatic intolerance	4	28.31 (10.58–75.73)	5 (3.24–6.08)	No
	VI^th^ nerve paralysis	4	36.99 (13.81–99.05)	5.38 (3.62–6.46)	No
	Guillain-Barre syndrome	3	4.74 (1.53–14.7)	2.59 (0.52–3.8)	No
	Intention tremor	3	49.87 (15.97–155.8)	5.86 (3.79–7.06)	No
	Monoplegia	3	4.55 (1.46–14.11)	2.54 (0.47–3.74)	No
	Post herpetic neuralgia	3	11.39 (3.67–35.39)	3.78 (1.71–4.99)	Uk (herpes zoster)
	Spinal cord compression	3	4.78 (1.54–14.83)	2.6 (0.53–3.81)	No
	Toxic encephalopathy	3	6.51 (2.1–20.22)	3.01 (0.95–4.22)	No
Psychiatric disorders	Delirium	15	3.63 (2.18–6.02)	2.08 (1.21–2.68)	Uk (mood altered)
	Listless	3	5.28 (1.7–16.38)	2.73 (0.66–3.94)	No

CI = Confidence Interval; FDA = Food and Drug Administration; IC = Information Component; ICANS = Immune effector Cell-Associated Neurotoxicity Syndrome; PT = Preferred Term; ROR = Reporting Odds Ratio; SOC = System Organ Class; and Uk = Unknown.

## Data Availability

This study was entirely based on publicly anonymized data made available by the Food and Drug Administration. The raw data can be downloaded at the following link: https://fis.fda.gov/extensions/FPD-QDE-FAERS/FPD-QDE-FAERS.html (accessed on 29 January 2024).

## Supplementary Materials

The following supporting information can be downloaded at https://www.mdpi.com/article/10.3390/ph17101266/s1: Table S1. Characteristics of neuropsychiatric mAb-related reports involving mAbs used for the treatment of myeloma multiple collected into FAERS. Table S2. Disproportionality analyses with ROR and IC for neuropsychiatric AEs related to mAbs approved for MM (including not signal).

## Abbreviations

ADCC	antibody-dependent cellular cytotoxicity
AE	adverse event
AMS	altered mental status
ATC	Anatomical Therapeutic Chemical
CI	confidence interval
FAERS	Food and Drug Administration Adverse Events Reporting System
FDA	Food and Drug Administration
GBS	Guillain-Barre syndrome
IC	information component
ICANS	immune effector cell-associated neurotoxicity syndrome
ICSR	Individual Case Safety Report
mAbs	monoclonal antibodies
MedDRA	Medical Dictionary for Regulatory Activities
MM	multiple myeloma
NDMM	newly diagnosed multiple myeloma
PN	peripheral neuropathy
PT	Preferred Term
ROR	Reporting Odds Ratio
RRMM	relapsed/refractory multiple myeloma
SDR	signal of disproportionate reporting
TTO	time to onset
VTE	vascular thromboembolic events
