# Supplementary material for: Neuropsychiatric Adverse Events with Monoclonal Antibodies Approved for Multiple Myeloma: An Analysis from the FDA Adverse Event Reporting System
Source: Pharmaceuticals (Basel). 2024 Sep 25;17(10):1266. doi: 10.3390/ph17101266 (PMC11510275; doi:10.3390/ph17101266)
Supplement: Supplementary file 1 [file pharmaceuticals-17-01266-s001.zip › pharmaceuticals-3214155-supplementary.pdf]

Neuropsychiatric Adverse Events with Monoclonal Antibodies Approved for Multiple Myeloma: An Analysis from the FDA Adverse Event Reporting System

Supplementary Materials

Table S1. Characteristics of neuropsychiatric mAb-related reports involving mAbs used for the treatment of myeloma multiple collected into FAERS.

Characteristic	Daratumumab (*n*= 2862)	Isatuximab (*n*= 345)	Elotuzumab (*n*= 321)	Belantamab Mafodotin (*n*= 241)	Teclistamab (*n*= 216)	Elranatamab (*n*= 29)	Talquetamab (*n*= 47)
**Age group, *n* (%)**							
Neonate	1 (0.03%)	2 (0.6%)					
Child	4 (0.1%)	3 (0.9%)					
Adolescent	10 (0.4%)						
Adult	719 (25.1%)	105 (30.4%)	87 (27.1%)	38 (15.8%)	43 (19.9%)	10 (34.5%)	4 (8.5%)
Elderly	1414 (49.4%)	191 (55.4%)	159 (49.5%)	69 (28.6%)	86 (39.8%)	15 (51.7%)	13 (27.7%)
Not available	714 (25.0%)	44 (12.8%)	75 (23.4%)	134 (55.6%)	87 (40.3%)	4 (13.8%)	30 (63.8%)
**Sex, *n* (%)**							
Female	1117 (39.0%)	156 (45.2%)	135 (42.1%)	73 (30.3%)	87 (40.3%)	10 (34.5%)	10 (21.3%)
Male	1341 (46.9%)	155 (44.9%)	172 (53.6%)	63 (26.1%)	83 (38.4%)	18 (62.1%)	17 (36.2%)
Not available	404 (14.1%)	34 (9.9%)	14 (4.4%)	105 (43.6%)	46 (21.3%)	1 (3.5%)	20 (42.6%)
**Primary source qualification, *n* (%)**							
Consumers	423 (14.8%)	14 (4.1%)	58 (18.1%)	69 (28.6%)	11 (5.1%)	3 (10.3%)	2 (4.3%)
Health professional	425 (14.9%)	134 (38.8%)	38 (11.8%)	19 (7.9%)	51 (23.6%)	8 (27.6%)	10 (21.3%)
Physician	1516 (53.0%)	169 (49.0%)	132 (41.1%)	144 (59.8%)	106 (49.1%)	17 (58.6%)	30 (63.8%)
Other health-professional	262 (9.2%)	17 (4.9%)	73 (22.7%)				
Pharmacist	222 (7.8%)	11 (3.2%)	20 (6.2%)	8 (3.3%)	45 (20.8%)	1 (3.5%)	5 (10.6%)
Not available	14 (0.5%)			1 (0.4%)	3 (1.4%)		
**Outcome codification, *n* (%)**							

Death	209 (7.3%)	21 (6.1%)	22 (6.9%)	52 (21.6%)	42 (19.4%)	3 (10.3%)	2 (4.3%)
Disability	46 (1.6%)	9 (2.6%)	5 (1.6%)	4 (1.7%)	5 (2.3%)		
Hospitalization - Initial or prolonged	946 (33.1%)	194 (56.2%)	102 (31.8%)	60 (24.9%)	65 (30.1%)	18 (62.1%)	12 (25.5%)
Life-threatening	82 (2.9%)	17 (4.9%)	10 (3.1%)		2 (0.9%)		1 (2.1%)
Other serious (Important Medical Event)	1366 (47.7%)	93 (27.0%)	131 (40.8%)	119 (49.4%)	76 (35.2%)	6 (20.7%)	10 (21.3%)
Required intervention to prevent permanent impairment/damage	1 (<0.1%)				4 (1.9%)		
Not available	212 (7.4%)	11 (3.2%)	51 (15.9%)	6 (2.5%)	22 (10.2%)	2 (6.9%)	22 (46.8%)
**Reporter Country, *n* (%)**							
Africa	19 (0.7%)						
Asia	504 (17.6%)	41 (11.9%)	74 (23.1%)	2 (0.8%)	6 (2.8%)	5 (17.2%)	3 (6.4%)
Central America	11 (0.4%)	7 (2.0%)					
Europe	1167 (40.8%)	211 (61.2%)	88 (27.4%)	120 (49.8%)	60 (27.8%)	10 (34.5%)	12 (25.5%)
North America	910 (31.8%)	65 (18.8%)	146 (45.5%)	109 (45.2%)	141 (65.3%)	13 (44.8%)	30 (63.8%)
Oceania	49 (1.7%)	8 (2.3%)	12 (3.7%)	5 (2.1%)	3 (1.4%)		
South America	159 (5.6%)	4 (1.2%)	1 (0.3%)	2 (0.8%)	5 (2.3%)	1 (3.5%)	
Not available	43 (1.5%)	9 (2.6%)		3 (1.2%)	1 (0.5%)		2 (4.3%)
**Year of reporting, *n* (%)**							
2015	8 (0.3%)	3 (0.9%)	6 (1.9%)				
2016	203 (7.1%)		48 (15.0%)				
2017	196 (6.9%)	2 (0.6%)	52 (16.2%)				
2018	350 (12.2%)	14 (4.1%)	50 (15.6%)				
2019	394 (13.8%)	16 (4.6%)	61 (19.0%)				
2020	326 (11.4%)	45 (13.0%)	60 (18.7%)	38 (15.8%)			
2021	374 (13.1%)	53 (15.4%)	30 (9.4%)	78 (32.4%)			
2022	517 (18.1%)	84 (24.4%)	11 (3.4%)	111 (46.1%)	20 (9.3%)	5 (17.2%)	
2023	494 (17.3%)	128 (37.1%)	3 (0.9%)	14 (5.8%)	196 (90.7%)	24 (82.8%)	47 (100.0%)
**Median age (Q1–Q3), years**	69 (61 - 76)	67 (61 - 74)	69 (60 - 76)	70 (61 - 76)	69 (60 - 75)	67 (59 - 75)	67 (65 - 71)
**Median weights (Q1–Q3), Kgs**	70 (59 - 84)	75 (62 - 89)	78 (60 - 92)	73 (62 - 82)	70 (59 - 82)	70 (58 - 79)	66 (53 - 79)

Table S2. Disproportionality analyses with ROR and IC for neuropsychiatric AEs related to mAbs approved for MM (including not signal).

Daratumumab						
**SOC**	**PT**	**N**	**ROR (95%CI)**	**Signal**	**IC (IC_{025}-IC_{075})**	**Signal**
**Nervous system disorders**	Neuropathy peripheral	533	5.89 (5.4-6.42)	Yes	2.64 (2.49-2.74)	Yes
	Polyneuropathy	189	17.74 (15.34-20.5)	Yes	4.15 (3.91-4.32)	Yes
	Dizziness	170	0.35 (0.3-0.41)	No	-0.22 (-0.48--0.04)	No
	Syncope	145	1.66 (1.41-1.95)	Yes	1.11 (0.83-1.31)	Yes
	Headache	134	0.2 (0.17-0.24)	No	-0.5 (-0.78--0.29)	No
	Tremor	126	0.86 (0.72-1.02)	No	0.45 (0.15-0.66)	Yes
	Hypoaesthesia	86	0.59 (0.48-0.73)	No	0.13 (-0.23-0.39)	No
	Loss of consciousness	80	0.77 (0.61-0.95)	No	0.35 (-0.02-0.61)	No
	Encephalopathy	71	5.07 (4.01-6.41)	Yes	2.48 (2.08-2.76)	Yes
	Cerebrovascular accident	61	0.5 (0.39-0.64)	No	0.01 (-0.42-0.31)	No
	Seizure	56	0.42 (0.32-0.55)	No	-0.11 (-0.55-0.21)	No
	Somnolence	53	0.29 (0.22-0.38)	No	-0.34 (-0.79--0.01)	No
	Paraesthesia	48	0.32 (0.24-0.42)	No	-0.28 (-0.76-0.06)	No
	Cerebral infarction	45	2.39 (1.79-3.21)	Yes	1.54 (1.05-1.9)	Yes
	Peripheral sensory neuropathy	45	10.49 (7.81-14.08)	Yes	3.45 (2.96-3.81)	Yes
	Depressed level of consciousness	42	1.65 (1.22-2.24)	Yes	1.12 (0.6-1.48)	Yes
	Cognitive disorder	36	0.8 (0.58-1.11)	No	0.39 (-0.16-0.79)	No
	Ischaemic stroke	33	2.24 (1.59-3.15)	Yes	1.47 (0.89-1.88)	Yes
	Altered state of consciousness	32	1.97 (1.39-2.78)	Yes	1.32 (0.73-1.74)	Yes
	Presyncope	32	1.42 (1-2.01)	Yes	0.96 (0.37-1.37)	Yes
	Lethargy	31	0.64 (0.45-0.91)	No	0.21 (-0.39-0.63)	No
	Neuralgia	31	1.13 (0.8-1.61)	No	0.72 (0.12-1.15)	Yes
	Posterior reversible encephalopathy syndrome	29	6.13 (4.25-8.84)	Yes	2.74 (2.12-3.18)	Yes
	Cerebral haemorrhage	28	0.93 (0.64-1.34)	No	0.53 (-0.1-0.98)	No
	Epilepsy	28	1.11 (0.77-1.61)	No	0.7 (0.07-1.15)	Yes
	Nervous system disorder	28	1.69 (1.16-2.45)	Yes	1.15 (0.52-1.59)	Yes
	Partial seizures	27	6.77 (4.63-9.89)	Yes	2.87 (2.23-3.33)	Yes
	Transient ischaemic attack	27	1.11 (0.76-1.61)	No	0.7 (0.06-1.16)	Yes
	Leukoencephalopathy	26	14.8 (10.04-21.84)	Yes	3.93 (3.28-4.4)	Yes

	Taste disorder	24	1.03 (0.69-1.54)	No	0.63 (-0.05-1.11)	No
	Guillain-Barre syndrome	23	6.42 (4.26-9.69)	Yes	2.81 (2.11-3.3)	Yes
	Spinal cord compression	23	6.48 (4.29-9.77)	Yes	2.82 (2.12-3.31)	Yes
	Brain oedema	20	2.51 (1.62-3.9)	Yes	1.62 (0.87-2.14)	Yes
	Disturbance in attention	20	0.4 (0.26-0.62)	No	-0.13 (-0.88-0.39)	No
	Dysgeusia	20	0.3 (0.19-0.46)	No	-0.31 (-1.06-0.21)	No
	Memory impairment	20	0.13 (0.08-0.2)	No	-0.66 (-1.41--0.14)	No
	Dementia	19	0.71 (0.45-1.11)	No	0.29 (-0.48-0.83)	No
	Facial paralysis	19	1.6 (1.02-2.52)	Yes	1.1 (0.33-1.64)	Yes
	Immune effector cell-associated neurotoxicity syndrome	18	5.36 (3.37-8.53)	Yes	2.58 (1.79-3.13)	Yes
	Peripheral sensorimotor neuropathy	18	22.42 (14.02-35.85)	Yes	4.51 (3.72-5.07)	Yes
	Neurotoxicity	17	1.69 (1.05-2.72)	Yes	1.16 (0.35-1.73)	Yes
	Coma	16	0.49 (0.3-0.8)	No	0.01 (-0.83-0.59)	No
	Amnesia	14	0.24 (0.14-0.41)	No	-0.41 (-1.32-0.21)	No
	Peripheral motor neuropathy	14	14.48 (8.53-24.58)	Yes	3.93 (3.02-4.55)	Yes
	Burning sensation	13	0.19 (0.11-0.33)	No	-0.52 (-1.46-0.13)	No
	Incoherent	12	2.61 (1.48-4.61)	Yes	1.68 (0.71-2.35)	Yes
	Balance disorder	11	0.12 (0.07-0.22)	No	-0.66 (-1.69-0.03)	No
	Speech disorder	11	0.23 (0.13-0.41)	No	-0.44 (-1.46-0.26)	No
	Unresponsive to stimuli	11	0.67 (0.37-1.21)	No	0.26 (-0.76-0.96)	No
	Dyskinesia	10	0.29 (0.15-0.53)	No	-0.32 (-1.4-0.4)	No
	Haemorrhage intracranial	10	0.82 (0.44-1.52)	No	0.44 (-0.64-1.17)	No
	Orthostatic intolerance	10	12.54 (6.71-23.43)	Yes	3.75 (2.67-4.48)	Yes
	Sensory disturbance	9	0.6 (0.31-1.15)	No	0.18 (-0.96-0.94)	No
	Dizziness postural	8	0.8 (0.4-1.6)	No	0.43 (-0.78-1.24)	No
	Stupor	8	4.48 (2.23-8.98)	Yes	2.39 (1.18-3.19)	Yes
	Aphasia	7	0.26 (0.13-0.55)	No	-0.35 (-1.66-0.5)	No
	Hemiparesis	7	0.52 (0.25-1.09)	No	0.08 (-1.22-0.93)	No
	Hepatic encephalopathy	7	1.04 (0.49-2.17)	No	0.69 (-0.62-1.54)	No
	Hypersomnia	7	0.24 (0.11-0.51)	No	-0.4 (-1.7-0.45)	No
	Facial paresis	6	2.14 (0.96-4.77)	No	1.49 (0.08-2.4)	Yes
	Haemorrhagic stroke	6	0.78 (0.35-1.75)	No	0.43 (-0.98-1.34)	No
	Hyperaesthesia	6	0.77 (0.34-1.71)	No	0.41 (-1-1.33)	No
	Intracranial mass	6	4.61 (2.07-10.3)	Yes	2.45 (1.04-3.36)	Yes
	Parkinsonism	6	1.01 (0.45-2.25)	No	0.67 (-0.74-1.59)	No
	Restless legs syndrome	6	0.34 (0.15-0.75)	No	-0.21 (-1.62-0.7)	No

	Senile dementia	6	10.24 (4.57-22.93)	Yes	3.52 (2.1-4.43)	Yes
	Ageusia	5	0.21 (0.09-0.51)	No	-0.45 (-2.01-0.54)	No
	Allodynia	5	8.64 (3.57-20.86)	Yes	3.31 (1.74-4.29)	Yes
	Cytotoxic oedema	5	41.17 (16.71-101.45)	Yes	5.44 (3.88-6.42)	Yes
	Dysaesthesia	5	1.9 (0.79-4.58)	No	1.37 (-0.19-2.36)	No
	Dysarthria	5	0.18 (0.07-0.42)	No	-0.53 (-2.09-0.46)	No
	Hyperammonaemic encephalopathy	5	7.57 (3.13-18.26)	Yes	3.13 (1.57-4.11)	Yes
	Mental impairment	5	0.23 (0.09-0.55)	No	-0.41 (-1.97-0.57)	No
	Paraparesis	5	3.69 (1.53-8.89)	Yes	2.18 (0.62-3.17)	Yes
	Parkinson's disease	5	0.25 (0.1-0.6)	No	-0.36 (-1.93-0.62)	No
	Sciatica	5	0.29 (0.12-0.69)	No	-0.29 (-1.85-0.69)	No
	Anosmia	4	0.38 (0.14-1.02)	No	-0.1 (-1.87-0.98)	No
	Central nervous system lesion	4	0.29 (0.11-0.76)	No	-0.28 (-2.05-0.8)	No
	Cerebellar haemorrhage	4	3.04 (1.14-8.11)	Yes	1.97 (0.2-3.04)	Yes
	Dystonia	4	0.42 (0.16-1.13)	No	-0.03 (-1.8-1.04)	No
	Formication	4	0.67 (0.25-1.79)	No	0.33 (-1.44-1.41)	No
	Language disorder	4	1.39 (0.52-3.7)	No	1.04 (-0.72-2.12)	No
	Metabolic encephalopathy	4	1.9 (0.71-5.08)	No	1.4 (-0.36-2.48)	No
	Monoparesis	4	2.45 (0.92-6.54)	No	1.7 (-0.06-2.78)	No
	Paralysis	4	0.33 (0.12-0.89)	No	-0.19 (-1.96-0.89)	No
	Paresis	4	2.46 (0.92-6.56)	No	1.7 (-0.06-2.78)	No
	Pleocytosis	4	11.42 (4.25-30.68)	Yes	3.72 (1.95-4.8)	Yes
	Post herpetic neuralgia	4	2.66 (1-7.11)	No	1.8 (0.04-2.88)	Yes
	Toxic encephalopathy	4	1.52 (0.57-4.06)	No	1.15 (-0.62-2.22)	No
	VIth nerve paralysis	4	6.48 (2.42-17.35)	Yes	2.95 (1.19-4.03)	Yes
	Autonomic neuropathy	3	3.93 (1.26-12.21)	Yes	2.34 (0.27-3.55)	Yes
	Brain fog	3	0.58 (0.19-1.81)	No	0.24 (-1.83-1.45)	No
	Cerebellar haematoma	3	11.42 (3.65-35.74)	Yes	3.77 (1.7-4.98)	Yes
	Chronic inflammatory demyelinating polyradiculoneuropathy	3	2.48 (0.8-7.69)	No	1.76 (-0.31-2.96)	No
	Dysstasia	3	0.1 (0.03-0.3)	No	-0.71 (-2.78-0.5)	No
	Encephalitis autoimmune	3	2.33 (0.75-7.24)	No	1.68 (-0.38-2.89)	No
	Focal dyscognitive seizures	3	1.76 (0.57-5.47)	No	1.35 (-0.72-2.56)	No
	Hypotonia	3	0.43 (0.14-1.32)	No	0 (-2.07-1.2)	No
	Loss of proprioception	3	12.5 (3.99-39.14)	Yes	3.89 (1.82-5.1)	Yes
	Migraine with aura	3	1.06 (0.34-3.29)	No	0.8 (-1.27-2)	No
	Motor dysfunction	3	0.32 (0.1-0.98)	No	-0.2 (-2.27-1.01)	No

	Neurological symptom	3	0.71 (0.23-2.2)	No	0.41 (-1.66-1.62)	No
	Parosmia	3	0.44 (0.14-1.37)	No	0.02 (-2.05-1.23)	No
	Toxic neuropathy	3	10.67 (3.41-33.38)	Yes	3.68 (1.61-4.88)	Yes
**Psychiatric disorders**	Confusional state	135	0.95 (0.81-1.13)	No	0.54 (0.26-0.75)	Yes
	Insomnia	105	0.4 (0.33-0.49)	No	-0.14 (-0.47-0.09)	No
	Delirium	54	2.29 (1.75-2.99)	Yes	1.49 (1.04-1.81)	Yes
	Anxiety	52	0.18 (0.13-0.23)	No	-0.56 (-1.02--0.23)	No
	Mental status changes	40	2.66 (1.95-3.63)	Yes	1.67 (1.14-2.04)	Yes
	Depression	34	0.17 (0.12-0.23)	No	-0.58 (-1.15--0.17)	No
	Agitation	18	0.33 (0.21-0.52)	No	-0.25 (-1.05-0.3)	No
	Sleep disorder	18	0.24 (0.15-0.38)	No	-0.42 (-1.22-0.13)	No
	Mental disorder	16	0.4 (0.25-0.66)	No	-0.13 (-0.97-0.46)	No
	Body dysmorphic disorder	15	58.08 (34.3-98.33)	Yes	5.81 (4.94-6.41)	Yes
	Disorientation	15	0.5 (0.3-0.83)	No	0.02 (-0.85-0.63)	No
	Hallucination	15	0.22 (0.13-0.37)	No	-0.45 (-1.32-0.15)	No
	Psychotic disorder	15	0.72 (0.43-1.2)	No	0.32 (-0.55-0.92)	No
	Aggression	13	0.37 (0.21-0.63)	No	-0.18 (-1.12-0.46)	No
	Anxiety disorder	12	3.6 (2.04-6.35)	Yes	2.08 (1.1-2.75)	Yes
	Depressed mood	12	0.21 (0.12-0.37)	No	-0.47 (-1.45-0.2)	No
	Personality change	12	1.58 (0.89-2.78)	No	1.1 (0.12-1.77)	Yes
	Irritability	8	0.15 (0.08-0.31)	No	-0.59 (-1.8-0.21)	No
	Restlessness	8	0.25 (0.13-0.5)	No	-0.38 (-1.59-0.42)	No
	Eating disorder	7	0.28 (0.14-0.6)	No	-0.31 (-1.62-0.54)	No
	Abnormal behaviour	6	0.21 (0.09-0.47)	No	-0.46 (-1.87-0.45)	No
	Listless	6	1.85 (0.83-4.13)	No	1.32 (-0.09-2.24)	No
	Panic attack	6	0.19 (0.08-0.42)	No	-0.5 (-1.92-0.41)	No
	Stress	6	0.07 (0.03-0.16)	No	-0.79 (-2.2-0.12)	No
	Mania	5	0.48 (0.2-1.14)	No	0.03 (-1.53-1.02)	No
	Nervousness	5	0.1 (0.04-0.24)	No	-0.71 (-2.27-0.28)	No
	Anger	4	0.14 (0.05-0.37)	No	-0.6 (-2.37-0.47)	No
	Hallucination, visual	4	0.25 (0.09-0.65)	No	-0.36 (-2.13-0.71)	No
	Mood altered	4	0.17 (0.06-0.44)	No	-0.54 (-2.31-0.54)	No
	Mood swings	4	0.14 (0.05-0.38)	No	-0.6 (-2.36-0.48)	No
	Poor quality sleep	4	0.17 (0.06-0.45)	No	-0.53 (-2.3-0.54)	No
	Affective disorder	3	0.41 (0.13-1.26)	No	-0.04 (-2.11-1.17)	No
	Apathy	3	0.24 (0.08-0.73)	No	-0.36 (-2.43-0.84)	No

	Bruxism	3	0.64 (0.21-1.98)	No	0.31 (-1.75-1.52)	No
	Emotional distress	3	0.02 (0.01-0.06)	No	-0.94 (-3.01-0.27)	No
	Major depression	3	0.49 (0.16-1.53)	No	0.11 (-1.96-1.31)	No
	Psychiatric symptom	3	0.44 (0.14-1.35)	No	0.01 (-2.06-1.22)	No
	Suicidal ideation	3	0.04 (0.01-0.13)	No	-0.87 (-2.94-0.34)	No
	Suicide attempt	3	0.07 (0.02-0.23)	No	-0.77 (-2.84-0.44)	No
**Belantamab Mafodotin**						
**SOC**	**PT**	**N**	**ROR (95%CI)**	**Signal**	**IC (IC_{025}-IC_{075})**	**Signal**
**Nervous system disorders**	Neuropathy peripheral	38	2.62 (1.9-3.61)	Yes	1.65 (1.11-2.03)	Yes
	Headache	22	0.21 (0.14-0.32)	No	-0.47 (-1.18-0.03)	No
	Dizziness	15	0.2 (0.12-0.33)	No	-0.5 (-1.37-0.1)	No
	Cerebrovascular accident	13	0.68 (0.39-1.17)	No	0.27 (-0.67-0.91)	No
	Syncope	13	0.94 (0.55-1.62)	No	0.56 (-0.37-1.21)	No
	Tremor	13	0.56 (0.33-0.97)	No	0.12 (-0.82-0.76)	No
	Cerebral haemorrhage	9	1.9 (0.99-3.67)	No	1.33 (0.19-2.09)	Yes
	Somnolence	9	0.31 (0.16-0.6)	No	-0.27 (-1.41-0.49)	No
	Cognitive disorder	8	1.14 (0.57-2.28)	No	0.77 (-0.44-1.58)	No
	Seizure	8	0.38 (0.19-0.77)	No	-0.14 (-1.35-0.67)	No
	Altered state of consciousness	6	2.35 (1.05-5.23)	Yes	1.6 (0.19-2.52)	Yes
	Burning sensation	6	0.56 (0.25-1.25)	No	0.15 (-1.27-1.06)	No
	Central nervous system lesion	5	2.29 (0.95-5.51)	No	1.59 (0.03-2.58)	Yes
	Encephalopathy	5	2.26 (0.94-5.44)	No	1.58 (0.01-2.56)	Yes
	Nervous system disorder	5	1.92 (0.8-4.62)	No	1.38 (-0.18-2.37)	No
	Neuralgia	5	1.16 (0.48-2.8)	No	0.83 (-0.73-1.82)	No
	Memory impairment	4	0.16 (0.06-0.44)	No	-0.55 (-2.31-0.53)	No
	Muscle tone disorder	4	59.56 (22.19-159.81)	Yes	6.06 (4.29-7.14)	Yes
	Paraesthesia	4	0.17 (0.06-0.45)	No	-0.53 (-2.3-0.55)	No
	Polyneuropathy	4	2.32 (0.87-6.19)	No	1.64 (-0.13-2.71)	No
	Aphasia	3	0.72 (0.23-2.23)	No	0.42 (-1.65-1.63)	No
	Bell's palsy	3	12.77 (4.11-39.68)	Yes	3.94 (1.87-5.15)	Yes
	Cerebral disorder	3	2.16 (0.69-6.69)	No	1.59 (-0.48-2.8)	No
	Lethargy	3	0.4 (0.13-1.23)	No	-0.05 (-2.12-1.15)	No
	Loss of consciousness	3	0.18 (0.06-0.57)	No	-0.49 (-2.56-0.72)	No
	Mental impairment	3	0.87 (0.28-2.71)	No	0.6 (-1.46-1.81)	No
	Neurological decompensation	3	12.49 (4.02-38.8)	Yes	3.91 (1.84-5.12)	Yes

	Sciatica	3	1.1 (0.35-3.42)	No	0.84 (-1.23-2.04)	No
	Subarachnoid haemorrhage	3	2.34 (0.75-7.27)	No	1.69 (-0.38-2.9)	No
	Unresponsive to stimuli	3	1.16 (0.37-3.6)	No	0.89 (-1.18-2.1)	No
**Psychiatric disorders**	Confusional state	21	0.95 (0.62-1.45)	No	0.56 (-0.18-1.07)	No
	Mental status changes	10	4.23 (2.28-7.88)	Yes	2.3 (1.22-3.03)	Yes
	Anxiety	6	0.13 (0.06-0.29)	No	-0.64 (-2.05-0.27)	No
	Agitation	4	0.47 (0.17-1.24)	No	0.03 (-1.73-1.11)	No
	Depressed mood	4	0.45 (0.17-1.2)	No	0.01 (-1.75-1.09)	No
	Sleep disorder	4	0.34 (0.13-0.9)	No	-0.18 (-1.95-0.89)	No
	Aggression	3	0.54 (0.17-1.67)	No	0.18 (-1.89-1.38)	No
**Elranatamab**						
**SOC**	**PT**	**N**	**ROR (95%CI)**	**Signal**	**IC (IC_{025}-IC_{075})**	**Signal**
**Nervous system disorders**	Altered state of consciousness	3	20.7 (6.61-64.83)	Yes	4.6 (2.53-5.81)	Yes
	Headache	3	0.51 (0.16-1.58)	No	0.14 (-1.93-1.34)	No
	Neuropathy peripheral	3	3.6 (1.15-11.28)	Yes	2.22 (0.15-3.42)	Yes
	Syncope	3	3.82 (1.22-11.98)	Yes	2.29 (0.22-3.5)	Yes
**Isatuximab**						
**SOC**	**PT**	**N**	**ROR (95%CI)**	**Signal**	**IC (IC_{025}-IC_{075})**	**Signal**
**Nervous system disorders**	Headache	24	0.17 (0.12-0.26)	No	-0.55 (-1.23--0.07)	No
	Syncope	23	1.26 (0.84-1.9)	No	0.84 (0.14-1.33)	Yes
	Polyneuropathy	21	9.26 (6.03-14.22)	Yes	3.31 (2.58-3.82)	Yes
	Cerebrovascular accident	17	0.67 (0.42-1.08)	No	0.25 (-0.56-0.82)	No
	Transient ischaemic attack	17	3.37 (2.09-5.42)	Yes	1.98 (1.17-2.55)	Yes
	Ischaemic stroke	14	4.59 (2.71-7.75)	Yes	2.39 (1.48-3.01)	Yes
	Dizziness	13	0.13 (0.07-0.22)	No	-0.65 (-1.59--0.01)	No
	Neuropathy peripheral	11	0.57 (0.31-1.02)	No	0.13 (-0.9-0.82)	No
	Peripheral sensory neuropathy	11	12.23 (6.76-22.13)	Yes	3.72 (2.7-4.42)	Yes
	Cerebral infarction	10	2.56 (1.38-4.76)	Yes	1.67 (0.59-2.4)	Yes
	Cerebral ischaemia	9	12.64 (6.56-24.34)	Yes	3.78 (2.64-4.54)	Yes
	Guillain-Barre syndrome	8	10.72 (5.35-21.48)	Yes	3.57 (2.35-4.37)	Yes
	Seizure	8	0.29 (0.15-0.58)	No	-0.3 (-1.52-0.5)	No

	Haemorrhage intracranial	7	2.76 (1.31-5.79)	Yes	1.79 (0.48-2.64)	Yes
	Altered state of consciousness	6	1.77 (0.8-3.95)	No	1.27 (-0.14-2.19)	No
	Basal ganglia infarction	6	132.39 (58.54-299.42)	Yes	7.11 (5.7-8.02)	Yes
	Peripheral motor neuropathy	6	29.61 (13.25-66.18)	Yes	5.01 (3.6-5.92)	Yes
	Presyncope	6	1.28 (0.57-2.85)	No	0.92 (-0.5-1.83)	No
	Aphasia	5	0.91 (0.38-2.18)	No	0.58 (-0.98-1.57)	No
	Paraesthesia	5	0.16 (0.07-0.38)	No	-0.56 (-2.12-0.42)	No
	Somnolence	5	0.13 (0.05-0.31)	No	-0.63 (-2.2-0.35)	No
	Subarachnoid haemorrhage	5	2.95 (1.23-7.09)	Yes	1.9 (0.34-2.89)	Yes
	Acute motor-sensory axonal neuropathy	4	93.02 (34.43-251.26)	Yes	6.68 (4.91-7.76)	Yes
	Cerebral haemorrhage	4	0.64 (0.24-1.7)	No	0.28 (-1.48-1.36)	No
	Generalised tonic-clonic seizure	4	1.36 (0.51-3.63)	No	1.02 (-0.74-2.1)	No
	Loss of consciousness	4	0.18 (0.07-0.49)	No	-0.5 (-2.26-0.58)	No
	Chronic inflammatory demyelinating polyradiculoneuropathy	3	11.95 (3.84-37.14)	Yes	3.85 (1.78-5.05)	Yes
	Dementia	3	0.54 (0.17-1.67)	No	0.17 (-1.9-1.38)	No
	Dysarthria	3	0.51 (0.16-1.57)	No	0.13 (-1.94-1.33)	No
	Epilepsy	3	0.57 (0.18-1.77)	No	0.22 (-1.85-1.43)	No
	Hepatic encephalopathy	3	2.14 (0.69-6.64)	No	1.58 (-0.49-2.79)	No
	Hypoaesthesia	3	0.1 (0.03-0.31)	No	-0.7 (-2.77-0.51)	No
	Meningoradiculitis	3	179.87 (56.31-574.58)	Yes	7.64 (5.57-8.85)	Yes
	Nervous system disorder	3	0.87 (0.28-2.7)	No	0.6 (-1.47-1.81)	No
	Posterior reversible encephalopathy syndrome	3	3.03 (0.98-9.41)	No	2.01 (-0.06-3.22)	No
	Sciatica	3	0.83 (0.27-2.58)	No	0.56 (-1.51-1.76)	No
	Sensory loss	3	1.86 (0.6-5.78)	No	1.42 (-0.65-2.62)	No
	Tremor	3	0.1 (0.03-0.3)	No	-0.7 (-2.77-0.51)	No
**Psychiatric disorders**	Insomnia	14	0.26 (0.15-0.43)	No	-0.38 (-1.28-0.24)	No
	Delirium	9	1.84 (0.95-3.53)	No	1.28 (0.15-2.05)	Yes
	Anxiety	7	0.11 (0.05-0.24)	No	-0.68 (-1.98-0.17)	No
	Confusional state	7	0.24 (0.11-0.5)	No	-0.4 (-1.71-0.45)	No
	Depression	7	0.17 (0.08-0.35)	No	-0.56 (-1.86-0.29)	No
	Acute psychosis	3	8.8 (2.83-27.34)	Yes	3.42 (1.36-4.63)	Yes
	Nervousness	3	0.29 (0.09-0.91)	No	-0.25 (-2.32-0.96)	No

Talquetamab						
**SOC**	**PT**	**N**	**ROR (95%CI)**	**Signal**	**IC (IC_{025}-IC_{075})**	**Signal**
**Nervous system disorders**	Dysgeusia	13	17.71 (10.11-31.02)	Yes	4.16 (3.22-4.8)	Yes
	Immune effector cell-associated neurotoxicity syndrome	7	185.55 (87.3-394.35)	Yes	7.59 (6.29-8.44)	Yes
	Taste disorder	7	26.81 (12.63-56.93)	Yes	4.82 (3.52-5.68)	Yes
	Ageusia	5	18.81 (7.75-45.66)	Yes	4.37 (2.81-5.36)	Yes
	Dizziness	3	0.54 (0.17-1.68)	No	0.18 (-1.89-1.39)	No
	Neurotoxicity	3	26.11 (8.35-81.62)	Yes	4.93 (2.86-6.14)	Yes

Teclistamab						
**SOC**	**PT**	**N**	**ROR (95%CI)**	**Signal**	**IC (IC_{025}-IC_{075})**	**Signal**
**Nervous system disorders**	Immune effector cell-associated neurotoxicity syndrome	96	450.7 (364.76-556.89)	Yes	8.66 (8.32-8.9)	Yes
	Neurotoxicity	22	31.49 (20.65-48.02)	Yes	5 (4.29-5.51)	Yes
	Headache	12	0.26 (0.15-0.45)	No	-0.37 (-1.35-0.3)	No
	Seizure	9	0.96 (0.5-1.86)	No	0.6 (-0.54-1.36)	No
	Neuropathy peripheral	8	1.21 (0.61-2.43)	No	0.84 (-0.37-1.64)	No
	Depressed level of consciousness	5	2.79 (1.16-6.71)	Yes	1.83 (0.27-2.82)	Yes
	Nervous system disorder	5	4.27 (1.78-10.28)	Yes	2.37 (0.81-3.36)	Yes
	Polyneuropathy	5	6.45 (2.68-15.53)	Yes	2.92 (1.36-3.91)	Yes
	Aphasia	4	2.14 (0.8-5.7)	No	1.54 (-0.23-2.61)	No
	Encephalopathy	4	4.02 (1.51-10.72)	Yes	2.32 (0.56-3.4)	Yes
	Hypersomnia	4	1.96 (0.73-5.23)	No	1.43 (-0.33-2.51)	No
	Spinal cord compression	4	15.87 (5.94-42.38)	Yes	4.19 (2.43-5.27)	Yes
	Tremor	4	0.38 (0.14-1.03)	No	-0.1 (-1.86-0.98)	No
	Unresponsive to stimuli	4	3.44 (1.29-9.19)	Yes	2.13 (0.36-3.21)	Yes
	Cerebral haemorrhage	3	1.41 (0.45-4.37)	No	1.1 (-0.97-2.3)	No
	Dysarthria	3	1.49 (0.48-4.64)	No	1.16 (-0.91-2.37)	No
**Psychiatric disorders**	Confusional state	9	0.9 (0.47-1.73)	No	0.54 (-0.6-1.3)	No

	Mental status changes	5	4.7 (1.95-11.31)	Yes	2.5 (0.94-3.48)	Yes
**Elotuzumab**						
**SOC**	**PT**	**N**	**ROR (95%CI)**	**Signal**	**IC (IC_{025}-IC_{075})**	**Signal**
**Nervous system disorders**	Neuropathy peripheral	41	2.52 (1.85-3.43)	Yes	1.6 (1.08-1.97)	Yes
	Syncope	27	1.75 (1.2-2.56)	Yes	1.19 (0.55-1.64)	Yes
	Cerebral infarction	25	7.61 (5.13-11.28)	Yes	3.03 (2.37-3.51)	Yes
	Dizziness	19	0.22 (0.14-0.35)	No	-0.45 (-1.22-0.09)	No
	Cerebrovascular accident	13	0.6 (0.35-1.04)	No	0.18 (-0.76-0.82)	No
	Tremor	13	0.5 (0.29-0.87)	No	0.04 (-0.9-0.68)	No
	Cerebral haemorrhage	12	2.27 (1.29-4)	Yes	1.52 (0.54-2.18)	Yes
	Hypoaesthesia	12	0.47 (0.27-0.83)	No	-0.01 (-0.99-0.65)	No
	Headache	11	0.09 (0.05-0.17)	No	-0.73 (-1.76--0.04)	No
	Somnolence	9	0.28 (0.14-0.53)	No	-0.33 (-1.47-0.43)	No
	Balance disorder	7	0.45 (0.22-0.95)	No	-0.02 (-1.32-0.83)	No
	Dementia	7	1.48 (0.71-3.11)	No	1.06 (-0.24-1.91)	No
	Seizure	7	0.3 (0.14-0.63)	No	-0.28 (-1.58-0.57)	No
	Memory impairment	6	0.22 (0.1-0.49)	No	-0.44 (-1.85-0.47)	No
	Lethargy	5	0.59 (0.25-1.42)	No	0.2 (-1.36-1.19)	No
	Loss of consciousness	5	0.27 (0.11-0.65)	No	-0.32 (-1.88-0.66)	No
	Cerebrovascular disorder	4	16.68 (6.24-44.56)	Yes	4.26 (2.5-5.34)	Yes
	Clumsiness	4	7.82 (2.93-20.86)	Yes	3.21 (1.45-4.29)	Yes
	Dysgeusia	4	0.34 (0.13-0.9)	No	-0.18 (-1.95-0.9)	No
	Neuralgia	4	0.83 (0.31-2.22)	No	0.52 (-1.24-1.6)	No
	Orthostatic intolerance	4	28.31 (10.58-75.73)	Yes	5 (3.24-6.08)	Yes
	VIth nerve paralysis	4	36.99 (13.81-99.05)	Yes	5.38 (3.62-6.46)	Yes
	Altered state of consciousness	3	1.05 (0.34-3.25)	No	0.78 (-1.29-1.99)	No
	Amnesia	3	0.29 (0.09-0.91)	No	-0.24 (-2.31-0.96)	No
	Burning sensation	3	0.25 (0.08-0.78)	No	-0.33 (-2.4-0.87)	No
	Central nervous system lesion	3	1.23 (0.4-3.8)	No	0.95 (-1.12-2.16)	No
	Depressed level of consciousness	3	0.67 (0.22-2.08)	No	0.36 (-1.71-1.57)	No
	Encephalopathy	3	1.21 (0.39-3.75)	No	0.93 (-1.14-2.14)	No

	Facial paralysis	3	1.44 (0.46-4.47)	No	1.12 (-0.95-2.33)	No
	Guillain-Barre syndrome	3	4.74 (1.53-14.7)	Yes	2.59 (0.52-3.8)	Yes
	Hemiparesis	3	1.27 (0.41-3.93)	No	0.98 (-1.09-2.19)	No
	Intention tremor	3	49.87 (15.97-155.8)	Yes	5.86 (3.79-7.06)	Yes
	Monoplegia	3	4.55 (1.46-14.11)	Yes	2.54 (0.47-3.74)	Yes
	Post herpetic neuralgia	3	11.39 (3.67-35.39)	Yes	3.78 (1.71-4.99)	Yes
	Spinal cord compression	3	4.78 (1.54-14.83)	Yes	2.6 (0.53-3.81)	Yes
	Toxic encephalopathy	3	6.51 (2.1-20.22)	Yes	3.01 (0.95-4.22)	Yes
	Transient ischaemic attack	3	0.7 (0.23-2.17)	No	0.4 (-1.67-1.6)	No
**Psychiatric disorders**	Insomnia	32	0.7 (0.49-0.99)	No	0.28 (-0.31-0.7)	No
	Delirium	15	3.63 (2.18-6.02)	Yes	2.08 (1.21-2.68)	Yes
	Confusional state	9	0.36 (0.19-0.69)	No	-0.18 (-1.32-0.58)	No
	Mental status changes	5	1.89 (0.78-4.53)	No	1.36 (-0.2-2.35)	No
	Agitation	3	0.31 (0.1-0.97)	No	-0.21 (-2.28-1)	No
	Anxiety	3	0.06 (0.02-0.18)	No	-0.81 (-2.88-0.39)	No
	Listless	3	5.28 (1.7-16.38)	Yes	2.73 (0.66-3.94)	Yes
	Mental disorder	3	0.43 (0.14-1.33)	No	0 (-2.07-1.21)	No

CI = Confidence Interval; FDA = Food and Drug Administration; IC = Information Component; ICANS = Immune effector Cell-Associated Neurotoxicity Syndrome; IRR= Immune Related Reaction; PT = Preferred Term; ROR = Reporting Odds Ratio; SOC = System Organ Class.
